# Pelvic Radiotherapy versus Radical Prostatectomy with Limited Lymph Node Sampling for High-Grade Prostate Adenocarcinoma

**DOI:** 10.1155/2016/2674954

**Published:** 2016-03-09

**Authors:** Christopher B. Baker, Andrew M. McDonald, Eddy S. Yang, Rojymon Jacob, Soroush Rais-Bahrami, Jeffrey W. Nix, John B. Fiveash

**Affiliations:** ^1^University of Alabama School of Medicine, Birmingham, AL 35233, USA; ^2^Department of Radiation Oncology, University of Alabama at Birmingham, Birmingham, AL 35249, USA; ^3^Department of Urology, University of Alabama at Birmingham, Birmingham, AL 35249, USA; ^4^Department of Radiology, University of Alabama at Birmingham, Birmingham, AL 35249, USA

## Abstract

*Purpose*. To compare oncologic outcomes for patients with Gleason score (GS) ≥ 8 prostate adenocarcinoma treated with radical prostatectomy (RP) versus external beam radiotherapy combined with androgen deprivation (RT + ADT).* Methods*. Between 2001 and 2014, 121 patients with GS ≥ 8 were treated at our institution via RT + ADT (*n* = 71) or RP (*n* = 50) with ≥ 1 year of biochemical follow-up. Endpoints included biochemical failure (BF), distant metastasis, and initiation of salvage ADT.* Results*. The RT + ADT group was older, had higher biopsy GS, and had greater risk of lymph node involvement. All other pretreatment characteristics were similar between groups. Mean number of lymph nodes (LNs) sampled for patients undergoing RP was 8.2 (±6.18). Mean biochemical follow-up for all patients was 61 months. Five-year estimates of BF for the RT + ADT and RP groups were 7.2% versus 42.3%, (*p* < 0.001). The RT + ADT group also had lower rates of distant metastasis (2% versus 7.8%) and salvage ADT (8% versus 33.8%).* Conclusion*. In this analysis, RT + ADT was associated with improved biochemical and metastatic control when compared to RP with limited LN sampling. How RT + ADT compares with more aggressive lymphadenectomy, as is currently our institutional standard, remains an important unanswered question.

## 1. Introduction

Patients with pretreatment PSA > 20, Gleason score (GS) ≥ 8, or T stage > 3a are classified as having high-risk prostate cancer [1]. Multiple randomized studies have shown that local disease control, via radical prostatectomy (RP) or radiation therapy (RT), improves survival outcomes for patients in the setting of high-risk disease compared to androgen deprivation therapy (ADT) alone [2, 3]. Two of the recommended initial treatments for high-risk disease are RT plus ADT (RT + ADT) or RP [1]. Among patients with high-risk disease, the presence of GS ≥ 8 has been associated with higher rates of disease progression and prostate cancer-specific mortality [4, 5]. There have been no randomized studies and only a few retrospective studies comparing the previously mentioned treatment modalities in patients with GS ≥ 8. The aim of the current study is to investigate and report the treatment outcomes of patients with high-grade (GS ≥ 8) prostate cancer treated with RT + ADT versus RP.

## 2. Methods and Materials

### 2.1. Inclusion Criteria

The records of all patients undergoing definitive external beam RT or RP for clinically localized prostate cancer at UAB since 2001 were reviewed. Due to the increased likelihood of metastatic disease on initial presentation, patients with an initial PSA (iPSA) > 50 ng/mL were omitted from this study. The remaining patients with GS ≥ 8 and at least 1 year of biochemical follow-up were included in the analysis. The defining GS could be via either transrectal biopsy or prostatectomy specimen. The University of Alabama at Birmingham Institutional Review Board approved this study.

### 2.2. Treatment Modalities

Patients were seen in consultation by both a radiation oncologist and urologist and underwent definitive RT or RP on the basis of patient and clinician preference after interdisciplinary discussion. Generally, patients with biopsy evidence of GS ≥ 8 were referred for RT due to the increased probability of occult lymph node (LN) involvement [6]. However, if a patient had a life expectancy ≥ 10 years and no serious comorbidities and the prostate was considered resectable, RP was also offered as a treatment option. This study specifically recorded incidences of preexisting coronary artery disease and diabetes for all patients.

Seventy-one patients were treated with definitive RT. Forty patients received dose-escalated conventionally fractionated RT to a total prostate dose of 75 to 77 Gy in 40 to 42 fractions and 31 patients received hypofractionated RT to a total prostate dose of 70 to 70.2 Gy in 28 fractions. All patients treated with definitive RT received elective nodal irradiation. Treatment was delivered via three-dimensional conformal RT or intensity-modulated RT. Neoadjuvant, concurrent, and adjuvant androgen deprivation therapy (ADT) was recommended to all patients undergoing definitive RT for a total duration of 24 months. One patient in the RT + ADT group also received taxane-based adjuvant chemotherapy as part of their initial definitive treatment regimen.

Fifty patients were initially treated with open retropubic, perineal, or robotic-assisted RP with or without pelvic LN dissection. Adjuvant postoperative RT was typically offered to patients whose pathologic specimen revealed adverse features (positive surgical margin, extracapsular extension, or seminal vesicle involvement) or whose PSA failed to become undetectable [1, 7] and was initiated after maximal recovery of urinary continence. Salvage RT was offered to patients whose PSA began to rise above 0.2 ng/mL after initially having been undetectable following surgery. For postoperative RT the prostate bed was prescribed 64.8 to 68.4 Gy and was always delivered at 1.8 Gy per fraction. Of the patients that received postoperative RT to the prostate bed, 10 patients also had pelvic LN irradiation. One patient in the RP group also received taxane-based adjuvant chemotherapy as part of their initial definitive treatment regimen.

### 2.3. Endpoint Definitions and Statistical Considerations

Patients returned for follow-up according to NCCN guidelines [1], including PSA measurement every 3 months for 2 years, every 6 months up to 5 years, and annually thereafter. Additional laboratory studies or imaging were obtained at the discretion of the treating physician on the basis of patient symptoms. The primary endpoint of this study was freedom from biochemical failure (BF). For definitive RT, BF was defined in accordance with the Radiation Therapy Oncology Group (RTOG) Phoenix consensus as an increase of 2 ng/mL above the PSA nadir [8]. For patients only treated with RP, BF was defined in accordance with the American Urology Association (AUA) guidelines as PSA ≥ 0.2 ng/mL followed by PSA > 0.2 ng/mL [9]. Lastly, if patients received salvage or adjuvant RT, then BF was defined as 2 consecutive PSA measurements ≥ 0.5 ng/mL after completion of RT [10]. To reduce bias against RP, patients who received postoperative salvage RT were not considered to have a BF until after RT. Secondary endpoints included freedom from distant metastasis and freedom from salvage ADT, with salvage ADT defined as ADT that was administered after the diagnosis of BF or distant metastasis.

Statistical analysis was performed utilizing IBM SPSS Statistics 22 software. Frequencies were compared using the Pearson Chi-square method and means were compared using the independent samples Mann-Whitney *U* test. Freedom from BF, distant metastasis, and salvage ADT was defined as the interval between the initial treatment date (surgery or RT) and most recent PSA/clinical follow-up or the date of the corresponding event. Death was not considered an event and patients who died during follow-up were censored from the analysis. Actuarial rates of BF, distant metastasis, and initiation of salvage ADT were calculated using the Kaplan-Meier method. Comparison of survival estimates was performed with the log-rank test.

## 3. Results

### 3.1. Pretreatment and Treatment Characteristics

**Figure 1:**
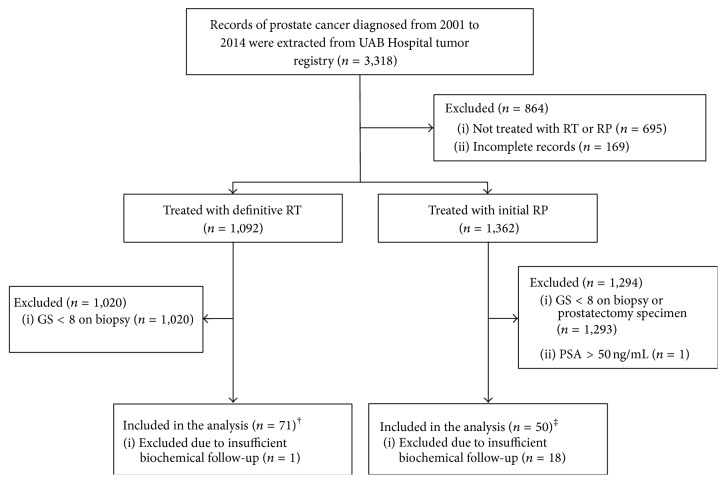
Consolidated Standards of Reporting Trials diagram. ^†^68/71 patients received neoadjuvant and/or adjuvant ADT; 1/71 received adjuvant chemotherapy. ^‡^22/50 patients received adjuvant RT; 12/50 received salvage RT; 1/50 received adjuvant chemotherapy.

A total of 3,318 patient records were extracted from the UAB Hospital tumor registry. Of these, 140 had clinically localized disease with GS ≥ 8. One patient was excluded due to iPSA > 50 ng/mL. Of the remaining patients, 121 had at least 1 year of clinical follow-up and were included in this analysis (Figure 1). Pretreatment and treatment characteristics are shown in Table 1. There was no statistically significant difference in clinical tumor stage, clinical node stage, or iPSA values between the two groups. Patients undergoing definitive RT had a higher risk of LN involvement based on Partin table risk stratification [6] (*p* = 0.006), and they also tended to be older than patients undergoing primary surgery, with a mean age of 69.93 versus 60.91 years, respectively (*p* < 0.001). There did not appear to be a difference between the two groups concerning underlying medical comorbidities.

Surgical and pathologic characteristics of the resection specimens for patients in the RP group are shown in Table 2. Thirty-eight patients had LNs sampled during surgery with a mean of 8.2 (±6.18) LNs sampled. Forty-four patients' prostatectomy specimen (88%) revealed adverse pathologic features and 22/44 of these patients received adjuvant postoperative RT. Twelve patients (27%) eventually were treated with salvage RT.

### 3.2. Treatment Outcomes

**Figure 2:**
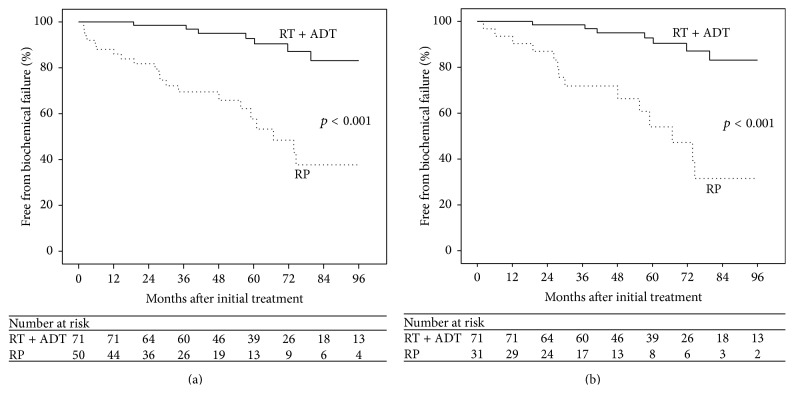
Kaplan-Meier estimate of freedom from BF in RT + ADT and RP patients with GS ≥ 8 on biopsy or pathology (a) and patients with GS ≥ 8 on biopsy (b).

The median biochemical follow-up for all patients was 61 months. The mean follow-up was slightly longer for patients undergoing definitive RT than for patients initially undergoing RP (73.7 months versus 60 months, *p* = 0.045). The Kaplan-Meier estimate of freedom from BF across all patients stratified by initial treatment modality is presented as in Figure 2(a). At 5 years, the rate of BF for patients initially treated with RT was 7.2% compared to 42.3% for patients initially treated with RP (*p* ≤ 0.001). A subset analysis of only patients with GS ≥ 8 on biopsy (*n* = 103) showed similar results and is presented as Figure 2(b). Five-year BF rates for RT (*n* = 71) and RP (*n* = 32) were 7.2% and 46%, respectively (*p* < 0.001).

**Figure 3:**
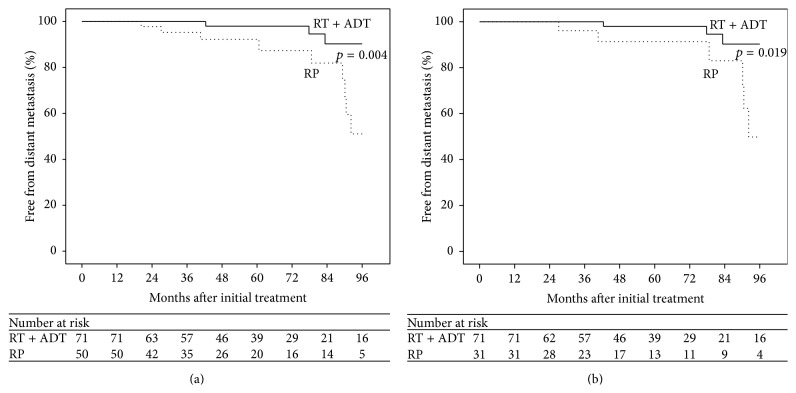
Kaplan-Meier estimate of freedom from distant metastasis in RT + ADT and RP patients with GS ≥ 8 on biopsy or pathology (a) and patients with GS ≥ 8 on biopsy (b).

The actuarial rates of distant metastasis at 5 years was 2% for patients who were initially treated with RT, compared to 7.8% for patients initially treated with RP (*p* = 0.004), as shown in Figure 3(a). Subset analysis of only patients with GS ≥ 8 on biopsy showed similar results and is presented in Figure 3(b). Five-year distant metastasis rates for RT and RP were 2% and 8.7%, respectively (*p* = 0.019).

**Figure 4:**
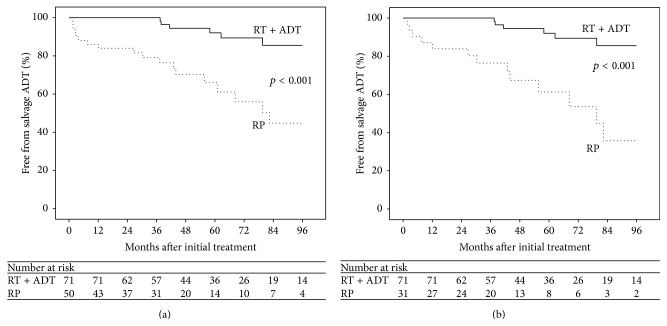
Kaplan-Meier estimate of freedom from salvage androgen deprivation therapy (ADT) in RT + ADT and RP patients with GS ≥ 8 on biopsy or pathology (a) and patients with GS ≥ 8 on biopsy (b).

Estimates of freedom from salvage ADT for all patients are shown in Figure 4(a). At 5 years, the rate of salvage ADT for all patients treated with definitive RT was 8%, compared to 33.8% for those initially treated with RP (*p* < 0.001). Subset analysis including only patients with GS ≥ 8 on biopsy showed similar results and is presented in Figure 4(b). Five-year salvage ADT rates for RT and RP were 8% and 38.7%, respectively (*p* < 0.001).

## 4. Discussion

The optimum treatment of high-risk localized prostate cancer has yet to be determined. The purpose of this study was to report the oncologic outcomes for patients with high-grade histology. Age at initial treatment, biopsy GS, and risk of LN involvement were weighted against the RT group. All other pretreatment characteristics between the two cohorts were well matched. Given the significant difference in age between the two cohorts, a comparison of overall survival would not have been meaningful. However, patients with GS ≥ 8 in our study who received RT + ADT showed improved rates of biochemical control and freedom from distant metastasis compared to those undergoing prostatectomy with limited LN sampling.

The 5-year rates of BF and distant metastasis that we observed in the RT + ADT group are lower than most of the previously published randomized or prospective studies [11–14]. Although there have been numerous studies investigating treatment outcomes of patients with localized prostate cancer, the vast majority of these studies have included patients with a wide range of Gleason scores. Even fewer studies have compared the biochemical and metastatic outcomes of patients with GS ≥ 8 disease treated with RP versus RT + ADT. Two retrospective studies have investigated outcomes of patients with GS ≥ 8 [15, 16]. Ramahi et al. reported 5-year distant metastasis and 5-year RP BF rates similar to our current study [15]. However, the 5-year BF rate for the RT group is more than twice our calculated rate of 7.2%. This discrepancy is most likely due to 95.8% of RT patients in our study receiving ADT, compared to 50.8% in the referenced study [15]. RT with 2-3 years of ADT is now a NCCN category 1 recommendation for patients with a high-risk of recurrence [1] based on randomized trial data showing decreased rates of clinical progression and increased overall survival compared to patients not receiving ADT [17]. A more recent study by Watkins et al. reported markedly higher 5-year BF rates of 79.4% for RP and 25.2% for RT + ADT, compared to the current study, and did not report metastatic outcomes [16]. These results are likely due a lower percentage of RP patients receiving adjuvant RT and a difference in the definition of BF for patients receiving salvage RT [16]. In contrast to the study by Watkins et al., patients in our study that received salvage RT were not scored as BF unless they experienced PSA relapse after completion of RT [16].

Treatment outcomes of definitive RT and RP are difficult to compare, given that the definition of BF differs between these two modalities and the role of adjuvant/salvage therapies following RP [7–9]. This remains a highly debated topic in regard to comparing treatment outcomes between RP and RT. Two notable studies have criticized and advised against comparing treatment outcomes of surgery and radiotherapy via their respective definitions of BF [18, 19]. Nielsen et al. analyzed the biochemical failure rates of 2,570 patients that underwent radical RP using the nadir +2 ng/mL and PSA ≥ 0.2 ng/mL definitions of biochemical failure. They concluded from this study that treatment outcomes between RT and RP should not be compared using these definitions, even though their study did not contain any patients treated with RT [19, 20]. A later study by Lee et al. stratified patients based on nomogram-predicted 5-year risk of BF and compared prostate cancer-specific mortality (PCSM) between RT and RP in patients treated between 1995 and 2008 [18, 21]. However, as the authors pointed out, the nomogram used to stratify RP patients utilized 2 consecutive measurements of PSA ≥ 0.4 ng/mL to define BF and treatment parameters used in RT patients may not reflect more current standard of care [18, 21]. Our current study uses the AUA recommended definition of 2 consecutive measurements of PSA ≥ 0.2 ng/mL in patients only treated with RP [9]. Both definitions of BF have been scrutinized and endorsed by AUA or ASTRO and have been shown to be predictors of disease progression [22, 23]. Given the importance of PSA measurements for disease screening, prognostication, and posttreatment monitoring, we believe our study makes an appropriate comparison between RT and RP. Furthermore, our current study includes two other endpoints (freedom from distant metastasis and freedom from salvage ADT) to confirm the biochemical outcomes of the two groups.

Approximately one-third of the RP patients in our study had GS < 8 on initial biopsy and, therefore, had GS ≥ 8 in their surgical specimen. A recent study demonstrated that MR/ultrasound fusion-guided biopsies could more accurately diagnose high-grade disease compared to traditional biopsies [24]. As discussed, results from our study indicate that patients with GS ≥ 8 may experience better disease control if initially treated with RT + ADT. If larger, prospective studies confirm that RT + ADT is the best initial treatment in the setting of high-grade prostate cancer, then targeted prostate biopsies could have an important role in selecting the best initial treatment for patients in this setting. There could also be quality-of-life implications with more accurate diagnosis of high-grade disease. Postoperative RT is commonly indicated in patients with high-grade disease. Conversely, if these patients were treated with RT + ADT initially, the potential adverse effects of RP and postoperative RT could be avoided. In brief, future research on the optimal treatment of high-grade prostate cancer and targeted biopsies could dramatically improve survival and toxicity outcomes in this patient population.

The retrospective nature and relatively small sample size are two inherent limitations in the current study. Another potential limitation of our study is the disproportionate use of neoadjuvant and/or adjuvant ADT within the RT group compared to RP patients, a potential lead time bias favoring RT. It is accepted that ADT can suppress PSA measurements during the androgen blockade, introducing a lead time bias in favor of patients treated with definitive RT. As part of the SWOG S9921 clinical trial, Dorff et al. reported that, upon completion of a 24-month course of ADT, testosterone levels normalized at a median time of 11.7 months [25]. Median duration of ADT in the RT group of our study was 24.3 months. Thus, we would expect to see an increased number of biochemical failures around the 36-month time point if ADT was “silencing” BF. However, this was not observed in our results, which had a median follow-up of 67 months for patients treated with RT + ADT. Additionally, numerous prospective studies have demonstrated the survival benefits of RT + ADT compared to RT monotherapy [26–28]. It is likely that RT + ADT truly results in superior biochemical and metastatic control versus RP and is not simply a byproduct of temporary PSA suppression in the setting of GS ≥ 8.

A previous study by Briganti et al. characterized the relationship between number of LNs removed during prostatectomy and the rate of nodal disease [29]. This study concluded that at least 10 LNs should be obtained for an adequate dissection [29]. Also, another recent study has suggested that removing more LNs in N1 patients is associated with increased prostate cancer-specific survival [30]. Patients in our study that underwent RP with pelvic LN sampling had a mean of 8.2 nodes sampled, which is inadequate according to the previously mentioned studies. Furthermore, current surgical guidelines recommend pelvic LN dissection in patients with high-risk prostate cancer seeking radical prostatectomy [1] and pelvic LNs cannot be accessed via the perineal approach alone (4% of the RP cohort). Additionally, 20% of the RP population underwent open retropubic or robotic radical prostatectomy without pathologic assessment of nodal status. This diversity of surgical approach and associated LN dissections is a limitation of our retrospective analysis of patients with adequate clinical follow-up.

Patients in the RT + ADT group may have died, and therefore censored, before experiencing an event, due to their increased age at treatment initiation. Nevertheless, larger, prospective, and randomized studies are needed to ultimately determine the optimum initial treatment in those with GS ≥ 8 prostate cancers. RTOG 0521 showed promising short-term results with an overall survival benefit in the chemotherapy arm for patients with high-risk disease [31]. As we await the long-term results of RTOG 0521, future trials should further investigate systemic therapy options as well.

## 5. Conclusion

When compared to RP with limited LN sampling, treatment of high-grade prostate cancer with RT + ADT was associated with improved biochemical and distant disease control and appeared to delay the need for salvage ADT. New targeted prostate biopsy techniques and other well-designed studies could lead to improved long-term outcomes in this population of high-risk prostate cancer patients.

## Figures and Tables

**Table 1:** Pretreatment and treatment characteristics.

Frequencies^1^ (%)	Definitive RT (*n* = 71)	Prostatectomy (*n* = 50)	*p* value
Biopsy Gleason score			
≤7	0	18 (37%)	<0.001
≥8	71 (100%)	31 (63%)	
Clinical T stage			
≤T2	63 (88.7%)	47 (96%)	0.162
T3	8^†^ (11.3%)	2^††^ (4%)	
Clinical N stage			
NX/N0	67 (94.4%)	49 (98%)	0.323
N1	4 (5.6%)	1 (2%)	
Neoadjuvant and/or adjuvant ADT			
Yes	68 (95.8%)	18 (36%)	<0.001
No	3 (4.2%)	32 (64%)	
Preexisting diabetes			
Yes	9 (12.7%)	10 (20%)	0.276
No	62 (87.3%)	40 (80%)	
Preexisting coronary artery disease			
Yes	15 (21.1%)	7 (14%)	0.317
No	56 (78.9%)	43 (86%)	

Means^2^ (range)			

Initial PSA:	9.58 (1.1–19.0)	11.52 (2.9–50.0)	0.350
Risk of LN involvement^‡^	13.02% (0–36.0%)	8.91% (0–36.0%)	0.006
Age at initial treatment	69.63 (50.44–83.61)	60.91 (42.43–75.34)	<0.001
Months of biochemical follow-up	73.74 (12.37–172.0)	60.03 (12.47–166.87)	0.045

^1^Pearson *χ* ^2^ test. ^2^Independent samples Mann-Whitney *U* test. *p* value < 0.05 is considered statistically significant.

^†^3 patients clinically classified as T3 via MRI; 4 via DRE; and 1 via CT.

^††^2 patients clinically classified as T3 via MRI.

^‡^Based on updated Partin tables nomogram [6].

NX: lymph nodes not sampled; N0: lymph nodes negative for disease; N1: lymph nodes positive for disease; RT: radiotherapy; PSA: prostate specific antigen.

**Table 2:** Surgical and pathologic characteristics of prostatectomy patients.

Frequencies (%)	Prostatectomy (*n* = 50)
Surgical approach^†^	
Perineal	2 (4%)
Retropubic	14 (28%)
Robotic	25 (50%)
Pathology Gleason score	
≤7	8 (16%)
≥8	42 (84%)
Pathologic T stage	
≤T2	13 (26%)
≥T3	37 (74%)
Pathologic node involvement	
NX	12 (24%)
N0	29 (58%)
N1	9 (18%)
Adverse pathology	
Positive margin	24 (49%)
SV invasion	22 (44%)
EC extension	37 (74%)
Any of the above	44 (88%)

Means (range)	

Nodes sampled	
N0	5.8 (1–17)
N1	15.71 (7–23)

EC: extracapsular; NX: lymph nodes not sampled; N0: lymph nodes negative for disease; N1: lymph nodes positive for disease; RT: radiotherapy; SV: seminal vesicle.

^†^Surgical approach data was unavailable for 9 patients.
